# Supplementary material for: A high-resolution mRNA expression time course of embryonic development in zebrafish
Source: eLife. 2017 Nov 16;6:e30860. doi: 10.7554/eLife.30860 (PMC5690287; doi:10.7554/eLife.30860)
Supplement: Supplementary file 6. [file elife-30860-supp6.zip › biolayout-clusters-files/Cluster111-genes.html]

Cluster111


# Cluster111: Genes

| Ensembl ID | Gene Name | Chr | Start | End | Biotype |
| --- | --- | --- | --- | --- | --- |
| ENSDARG00000012341 | capn9 | 1 | 53222101 | 53247349 | protein\_coding |
| ENSDARG00000014522 | cdh6 | 2 | 28393410 | 28542893 | protein\_coding |
| ENSDARG00000007347 | degs1 | 13 | 371939 | 384552 | protein\_coding |
| ENSDARG00000027750 | dpp7 | 5 | 28559758 | 28577119 | protein\_coding |
| ENSDARG00000075041 | fmnl2b | 6 | 13156750 | 13215514 | protein\_coding |
| ENSDARG00000034943 | si:ch211-127l15.5 | 14 | 31525010 | 31543783 | protein\_coding |
| ENSDARG00000093760 | si:ch211-197h24.9 | 16 | 27669116 | 27674549 | protein\_coding |
| ENSDARG00000086874 | si:ch211-232i5.3 | 20 | 8093970 | 8106979 | protein\_coding |
| ENSDARG00000031911 | tns3.2 | 20 | 6561364 | 6676694 | protein\_coding |
| ENSDARG00000073723 | zgc:194679 | 7 | 56375648 | 56381503 | protein\_coding |
| ENSDARG00000078550 | zgc:194839 | 8 | 32792603 | 32795982 | protein\_coding |
